# β-Caryophyllene protects against ischemic stroke by inhibiting H3K9 and H3K18 lactylation-mediated cellular pyroptosis

**DOI:** 10.3389/fphar.2026.1840441

**Published:** 2026-06-17

**Authors:** Qing Xin, Fei Xu, Jie Wu

**Affiliations:** 1 Department of Physiology, and Institute of Brain Science and Diseases, School of Basic Medicine, Qingdao University, Qingdao, China; 2 Department of Physiology, Jining Medical University, Jining, China; 3 Department of Vascular Surgery, Jining NO.1 People’s Hospital, Jining, China; 4 Department of Pharmacology, Shantou University Medical College, Shantou, China

**Keywords:** H3K18 lactylation, H3K9 lactylation, ischemic stroke, pyroptosis, β-caryophyllene

## Abstract

**Background:**

Ischemic stroke is a common and severe cerebrovascular disease with high mortality and disability. Accumulating evidence indicates that β-caryophyllene (BCP) exerts neuroprotective effects against cerebral ischemic injury; however, the precise underlying mechanisms remain largely unexplored.

**Methods:**

Focal cerebral ischemia/reperfusion (I/R) mouse models were established *in vivo* and oxygen-glucose deprivation/reoxygenation (OGD/R) was conducted in BV2 microglial cells and primary microglia *in vitro*.

**Results:**

We demonstrated that BCP administration significantly reduced cerebral infarct volume, alleviated neurological deficits, and enhanced motor function in mice subjected to transient focal cerebral ischemia. Mechanistically, BCP inhibited pyroptosis and glycolysis in the ischemic penumbra of mice and in BV2 cells following OGD/R. Concomitantly, BCP decreased the levels of H3K9 lactylation (H3K9la) and H3K18 lactylation (H3K18la) in brain tissues of ischemic penumbra and in OGD/R-induced BV2 cells. Notably, co-treatment with lactate attenuated these inhibitory effects and abrogated the neuroprotective efficacy of BCP. Similar results were also obtained in primary microglia. Additionnaly, oxamate (the LDHA inhibitor) simultaneously downregulated the protein levels of H3K9la, H3K18la, and pyroptosis-related factors, while MCC950 (the NLRP3 inflammasome inhibitor) only blocked downstream pyroptosis without affecting histone lactylation. Chip-PCR further demonstrated that OGD/R increased the enrichment of H3K9la and H3K18la at the NLRP3 promoter, which was decreased by BCP and oxamate but not by MCC950. Lactate supplementation partially restored the inhibitory effects of BCP.

**Conclusion:**

BCP protects against ischemic stroke by targeting the lactate-histone lactylation-pyroptosis axis, providing a potential therapeutic target for cerebral ischemia.

## Introduction

Ischemic stroke is a major cerebrovascular disease, which seriously threatens people’s lives and health. Intravenous thrombolysis and mechanical thrombectomy are the main treatments for acute ischemic stroke, aiming to restore cerebral blood flow (CBF) in the ischemic area as soon as possible (Furie and Kelly, 2026). However, the restoration of blood flow can paradoxically lead to further tissue and microcirculatory damage, a phenomenon known as ischemia/reperfusion (I/R) injury. Multiple pathological mechanisms, including energy metabolism disorder, oxidative stress, and inflammation are implicated in the pathogenesis of cerebral I/R (Liu et al., 2024). Among them, inflammation is reported to be closely associated with the occurrence, development, and prognosis of ischemic stroke. Pyroptosis, a unique form of inflammatory programmed necrosis, is characterized by cell swelling, rupture, and release of proinflammatory contents (Lin et al., 2023). Inflammasomes, specifically the NLRP3, play essential regulatory roles in the process of pyroptosis (Wu et al., 2022). The NLRP3 inflammasome is a multi-molecule complex mainly composed of a sensor protein NLRP3, an adaptor protein ASC, and an effector protein pro-caspase1 (Wang et al., 2022a). Upon activation, the NLRP3 inflammasome triggers the autoactivation of pro-caspase1, which subsequently mediates the maturation and secretion of pro-inflammatory cytokines (IL-1β and IL-18) and cleaves gasdermin D (GSDMD), ultimately culminating in pyroptosis (Bellut et al., 2023).

Ischemic stroke often induces a metabolic shift from oxidative phosphorylation (OxPhos) to glycolysis, resulting in a significant accumulation of lactate within the brain. Accumulating evidence suggests that lactate acts not merely as a glycolytic byproduct but also serves as a critical signaling molecule and a substrate for protein lactylation, influencing various biological processes (Fang et al., 2024; Wang M. Y. et al., 2024). Increased lactate could facilitate lactylation of histone lysine residues (histone lactylation), which functions as a novel posttranslational modification that directly regulates gene expression (Zhang et al., 2019). Numerous studies have subsequently demonstrated that histone lactylation modulates multiple cellular, physiological, and pathological processes, including immune response (Ziogas et al., 2025) and many neurological diseases (Wang et al., 2024d).

Among the various lactylation sites, lactylation of histone H3 at lysine residue 9 (H3K9la) and lysine residue 18 (H3K18la) have garnered increasing attention. For instance, under hypoxic conditions, H3K9la is upregulated in head and neck squamous cell carcinoma cells and promotes IL-11 transcription (Wang R. et al., 2024). In the context of cerebral ischemia, H3K9la—identified as the most upregulated histone lactylation site in post-ischemic microglia—drives glycolytic reprogramming and pro-inflammatory M1 polarization by promoting the transcription of LDHA and HIF-1α (Si et al., 2024). Similarly, elevated H3K18la levels have been observed in sepsis-associated acute kidney injury, where it activates NF-κB signaling to exacerbate inflammation and renal dysfunction (Qiao et al., 2024). Furthermore, increased H3K18la in senescent microglia and hippocampal tissues of aged and Alzheimer’s disease (AD) models stimulates NF-κB signaling to induce a senescence-associated secretory phenotype (Wei et al., 2023). Notably, the levels of H3K18la were upregulated in bilirubin-stimulated primary astrocytes and the hippocampus of bilirubin encephalopathy rats, and its mediation of nucleotide-binding oligomerization domain-2 (NOD2) expression promoted pyroptosis in primary astrocytes exposed to bilirubin (Li et al., 2025). A recent study further indicated that H3K18la might promote astrocytic neuroinflammation and exacerbate cerebral I/R injury (Wang et al., 2025). Together, these studies suggest that H3K9la and H3K18la might play pro-inflammatory roles; however, the specific involvement of these two histone lactylation sites in ischemic stroke-induced cellular pyroptosis remains to be elucidated.

β-Caryophyllene (BCP), a natural bicyclic sesquiterpene classified as a phytocannabinoid and a selective agonist of the cannabinoid receptor 2, has exhibited multiple biological properties, including anti-inflammatory, antioxidant, and neuroprotective effects, and thus holds broad application prospects in Parkinson’s disease (PD), stroke and other central nervous system (CNS) diseases (Brand-Rubalcava et al., 2023; Ricardi et al., 2025). Furthermore, our previous research revealed that BCP could alleviate ischemic white matter injury by inhibiting oligodendrocyte pyroptosis (Xin et al., 2024). Despite these advances, the precise molecular targets through which BCP exerts its neuroprotective effects remain incompletely understood. While the link between histone lactylation and pyroptosis is emerging, it remains unknown whether BCP confers neuroprotection by modulating specific histone lactylation sites, particularly H3K9la and H3K18la. Based on these considerations, the present study aimed to investigate the role of H3K9la and H3K18la in cellular pyroptosis and to clarify whether they mediate the neuroprotective effects of BCP in a mouse model of cerebral ischemia.

## Materials and methods

### Drugs and chemicals

β-Caryophyllene (BCP), sodium lactate, oxamate and MCC950 were purchased by Sigma-Aldrich (United States). Antibodies for HK2 and CD11 b were obtained from Abcam (United States). Antibodies for NLRP3, GSDMD, PFKFB3 and Cleaved caspase1 were supplied by Cell Signaling Technology (United States). Antibodies for β-Actin was procured by Proteintech Group (China). Antibodies for H3K9la and H3K18la were acquired from PTM BIO (China).

### Animals

All the experimental procedures and protocols involving animals were reviewed and approved by the Animal Care and Use Committee of Jining Medical University prior to the commencement of the study. Animal care and experimental operations were conducted in strict accordance with the *Guide for the Care and Use of Laboratory Animals* published by the National Research Council. Male C57BL/6 J mice (9 weeks old, weighing 26–30 g) were obtained from Jinan Pengyue Experimental Animal Breeding Co., Ltd (Jinan, China). Mice were housed in a controlled environment under a 12-h light/dark cycle with *ad libitum* access to food and water. All behavioral assessments and analyses were conducted by investigators blinded to the experimental groups.

### Cerebral I/R model and experimental groups

The focal cerebral I/R mouse model was established using a modified intraluminal filament technique as previously described (Wang et al., 2022b). Briefly, mice were anesthetized with 1.25% 2,2,2-tribromoethanol (10 mL/kg, intraperitoneally) and maintained on a thermostatic heating pad to keep the body temperature at 37 °C. Under a surgical microscope, the right common carotid artery (CCA), external carotid artery (ECA), and internal carotid artery (ICA) were exposed via a ventral midline neck incision. A nylon monofilament (0.22–0.23 mm in diameter; RWD Life Science, Shenzhen, China) was inserted into the ECA lumen and gently advanced into the ICA until it occluded the origin of the middle cerebral artery (MCA). After 90 min of occlusion, the filament was withdrawn to allow reperfusion. Regional cerebral blood flow (CBF) was monitored using laser Doppler flowmetry; only mice exhibiting a CBF reduction to <20% of baseline during occlusion were included in the study. Mice were randomly assigned to the following experimental groups: (1) Sham, (2) I/R, (3) I/R + BCP, and (4) I/R + lactate + BCP. The Sham group underwent the same surgical procedure without MCA occlusion. The I/R group was subjected to 90 min of ischemia followed by 24 h of reperfusion and received an intracerebroventricular (ICV) injection of 2 μL sterile saline and an intraperitoneal (i.p.) injection of 200 μL vehicle (corn oil containing 1% DMSO) at 30 min before ischemia and immediately upon reperfusion. The I/R + BCP group received an i. p. injection of 200 μL BCP (50 mg/kg, dissolved in corn oil containing 1% DMSO) at both time points. The I/R + lactate + BCP group received an ICV injection of 2 μL sodium lactate (100 mmol/L) and an i. p. injection of 200 μL BCP (50 mg/kg) at both time points. Neurological function and infarct volume were evaluated at 24 h post-reperfusion.

### Intracerebroventricular injection

Mice were anesthetized with 1.25% 2,2,2-tribromoethanol and positioned in a stereotaxic apparatus. Following a scalp incision, a burr hole was drilled at the coordinates 1.0 mm lateral and 0.46 mm posterior to bregma. A 5-μL Hamilton syringe was lowered to a depth of 2.5 mm, and either sodium lactate or sterile saline was administered at a rate of 0.5 μL/min. The needle was left in place for an additional 5 min post-injection to prevent backflow.

### Neurological deficit measurement

Neurological deficits were evaluated using the modified Neurological Severity Score (mNSS) as previously described (Chen et al., 2001). The mNSS comprises motor, sensory, reflex, and balance tests, yielding a composite score ranging from 0 (no deficit) to 18 (severe deficit). All assessments were performed by an investigator blinded to the treatment groups.

### Rotarod test

Motor coordination and balance was assessed using the accelerating rotarod test (Ehinger et al., 2021). Mice were trained on the rotarod apparatus (Ugo Basile, Italy) for three consecutive days prior to surgery. The rod rotation speed was gradually increased from 4 to 40 rpm over 5 min. Latency to fall was recorded for each mouse. Baseline performance was determined by averaging three trials on the day preceding surgery. Data are expressed as the percentage of the mean trial duration relative to the individual baseline value.

### Evaluation of cerebral infarction

At 24 h post-reperfusion, mice were euthanized, and brains were rapidly removed and sectioned into five 2-mm-thick coronal slices using a mouse brain matrix. The slices were stained with 2% 2,3,5-triphenyltetrazolium chloride (TTC) solution at 37 °C for 30 min in the dark and subsequently fixed in 4% paraformaldehyde. Infarct areas were quantified using ImageJ software (NIH, Bethesda, MD, United States).

### Immunofluorescence analysis

Brain tissue sections, BV2 cells, or primary microglia were fixed and permeabilized, then blocked with 5% bovine serum albumin (BSA; Solarbio Life Sciences, China) at room temperature (RT) for 1 h. Samples were incubated overnight at 4 °C with primary antibodies against H3K9la or H3K18la (1:100, PTM BIO). After three washes with phosphate-buffered saline (PBS), sections were incubated with Alexa Fluor 488-conjugated goat anti-rabbit IgG (Proteintech Group, China) for 1 h at RT. Nuclei were counterstained with 4′,6-diamidino-2-phenylindole (DAPI). Three fields were selected randomly to capture the images using an inverted fluorescence microscope (Olympus, Tokyo, Japan). The mean fluorescence intensity in different groups was analyzed using ImageJ software. Four biological replicates for each sample group were included.

### Cell culture and treatments

The murine microglial cell line BV2 was obtained from Procell (Wuhan, China) and cultured in high-glucose DMEM (Gibco, United States) supplemented with 10% FBS and 1% penicillin-streptomycin in a humidified incubator at 37 °C with 5% CO_2_. Primary mouse microglia were prepared as previously described (Nissen and Tsirka, 2016; Guan et al., 2025). Neonatal C57BL/6 mice (within 48 h of birth) were euthanized. Under sterile conditions, brains were harvested and placed in prechilled HBSS under an anatomical microscope. The meninges and blood vessels were carefully stripped, and the cerebral cortex was isolated, minced into small pieces and digested with trypsin for 10 min in a 37 °C water bath shaker. The digestion was terminated by the addition of DMEM medium containing 5% fetal bovine serum and 1% penicillin-streptomycin. After digestion, the cell suspension was filtered through 100-mesh, 200-mesh and 400-mesh sieves and centrifuged. Following centrifugation, the supernatant was discarded, and the cell pellet was resuspended with mouse microglia cell complete medium (Procell, Wuhan) and seeded in Poly-L-lysine-coated dishes. Thereafter, cells were left undisturbed for 3 days at 37 °C in a 5% CO_2_ incubator. On the third day of culture, the old medium was removed and replaced with fresh media. Cultures were maintained continuously until day 9 without additional medium replacement. The culture medium was aspirated from the dishes, and 4 mL of lidocaine hydrochloride (12 mM, pH 7.4) was added, followed by incubation at 37 °C for 5 min. The dishes were then flicked gently for another 5 min to allow sufficient detachment and suspension of microglia. The supernatant containing suspended cells was collected, centrifuged, resuspended and reseeded in culture dishes, followed by a further 24 h of static incubation at 37 °C with 5% CO_2_. Cells were allowed to adhere for 24 h, followed by medium refreshment, and the cells were then available for subsequent experiments. The purity of primary microglia was assessed by immunofluorescence staining of CD11 b. The entire experiment strictly followed the requirements of aseptic operation to avoid any contamination and impairment of cell viability. To induce oxygen-glucose deprivation/reoxygenation (OGD/R), cells were washed twice with PBS and incubated in glucose-free DMEM before being placed in a hypoxic chamber (95% N_2,_ 5% CO_2_) for 6 h. Reoxygenation was initiated by replacing the medium with standard DMEM containing 10% FBS and returning the cells to normoxic conditions (95% O_2_, 5% CO_2_) for 24 h. Control cells were maintained in normal high-glucose DMEM under normoxic conditions. For drug intervention, cells were pretreated with 1 μM BCP, 20 mM sodium lactate, 20 mM oxamate or 1 μM MCC950 for 24 h prior to OGD/R exposure.

### Western blotting

Brain tissues, BV2 cells or primary microglia were lysed in RIPA buffer (Beyotime, Shanghai, China) containing protease inhibitors on ice. Lysates were centrifuged at 12,000 rpm for 30 min at 4 °C, and supernatant protein concentrations were determined using a BCA Protein Assay Kit (CWBIO, Beijing, China). Equal amounts of protein were separated by 10% or 12% SDS-PAGE gels and transferred onto polyvinylidene difluoride (PVDF) membranes (Millipore, Bedford, MA, United States). Membranes were blocked with 5% BSA in Tris-buffered saline containing 0.1% Tween-20 (TBST) for 2 h at RT, followed by overnight incubation at 4 °C with primary antibodies against NLRP3, GSDMD, cleaved caspase-1, HK2, PFKFB3, H3K9la, H3K18la, and β-actin. After washing with TBST, membranes were incubated with horseradish peroxidase (HRP)-conjugated secondary antibodies for 1 h at RT. Protein bands were visualized using an enhanced chemiluminescence (ECL) detection system (Proteintech Group, China), and band intensities were quantified using ImageJ software. The Western blotting experiments were repeated independently for four times. The relative protein expression was normalized to β-actin.

### Measurement of lactate content

To measure the content of lactate, the supernatants were collected from isolated brain tissues, digested BV2 cells or primary microglia in each experimental group, and the lactate assay kit (Jiancheng, China) was used for measuring lactate levels according to the manufacturer’s instructions. Absorbance was measured at 530 nm using a spectrophotometer.

### Transmission electron microscopy

For transmission electron microscopy (TEM), cells were fixed in 2.5% glutaraldehyde at 4 °C for 24 h. After fixation, samples were post-fixed with 1% osmium tetroxide, dehydrated through a graded series of ethanol, and embedded in epoxy resin. Ultrathin sections (70 nm) were cut using an ultramicrotome (UC7, Leica, Wetzlar, Germany), stained with uranyl acetate and lead citrate, and examined using a TEM (Hitachi H-7650, Tokyo, Japan).

### Chip-PCR

The Chip-PCR assay was performed according to the manufacturer’s instructions (Beyotime, P2080S). The main steps are briefly outlined below. Primay micoglial cells were fixed with 1% formaldehyde at 37 °C for 10 min and then incubated with glycine solution for 5 min. The fixed cells were resuspended in SDS lysis buffer containing protease inhibitors. Then, the chromatin was processed by ultrasonic fragmentation to obtain approximately 200–1,000 bp fragments. After 2% of diluted chromatin was kept as the input, the remaining samples were incubated with rabbit anti-H3K9la or anti-H3K18la antibodies overnight at 4 °C with rotation. Normal IgG was used to determine non-specific bindings. The above mixtures were next incubated with Protein A/G Magnetic Beads for 60 min on a shaker at 4 °C, and the protein-DNA complexes were eluted after washes. The samples were incubated at 65 °C for 4 h and digested with proteinase K. Finally, the eluted DNA was quantified by SuperStar Universal SYBR Master Mix and the Roche LC480 real-time PCR (RT-PCR) system. The primer sequences of NLRP3 promoter region are shown in Table 1.

**TABLE 1:** Primers used for ChIP-PCR targeting the NLRP3 promoter region.

Primer name	Primer sequence (5′-3′)	Product size (bp)
NLRP3 promoter	Forward: TCTTGGGCACGCTCAGTAAG Reverse: TGGCAGAGACTCCAACCAAC	210

### Statistical analysis

Data were analyzed using GraphPad Prism software (version 9.0; GraphPad Software, San Diego, CA, United States). Statistical comparisons between two groups were performed using an unpaired Student’s t-test. Differences among multiple groups were assessed using one-way analysis of variance (ANOVA) followed by Tukey’s *post hoc* test. Data are expressed as the mean ± standard deviation (SD). A p-value of <0.05 was considered statistically significant.

## Results

### BCP reduces cerebral infarct volume and neurological deficits after I/R

**FIGURE 1:**
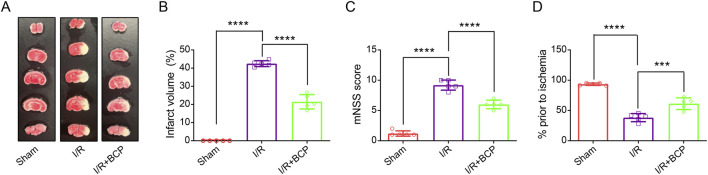
Treatment with BCP decreased the infarct volume and neurological deficits in mice with ischemic stroke. **(A)** Representative images of TTC staining in each group; **(B)** Quantitative analysis of the infarct volume; **(C)** Analysis of mNSS scores in each group; **(D)** Analysis of rotarod performance in each group. Data are expressed as mean ± SD (n = 5). ^***^P < 0.001; ^****^P < 0.0001.

To evaluate the neuroprotective efficacy of β-caryophyllene (BCP), we first assessed infarct volume and neurological function following focal cerebral ischemia/reperfusion (I/R). Triphenyltetrazolium chloride (TTC) staining revealed a marked increase in infarct volume in the I/R group compared to sham-operated controls, an effect that was significantly attenuated by pretreatment with BCP (Figures 1A,B). Consistent with these findings, mice subjected to I/R exhibited significantly higher modified Neurological Severity Scores (mNSS) compared to sham mice, and this deficit was significantly improved by BCP treatment (Figure 1C). Furthermore, in the accelerating rotarod test, while sham-operated mice maintained stable performance, I/R-exposed mice showed a significant decline in latency to fall at 24 h post-surgery. Notably, mice in the I/R + BCP group demonstrated significantly better motor coordination and balance compared to the I/R group (Figure 1D). Collectively, these data demonstrate that BCP effectively reduces infarct size and improves behavioral outcomes following ischemic stroke.

### BCP suppresses cellular pyroptosis in mice with ischemic stroke and OGD/R-treated BV2 cells

**FIGURE 2:**
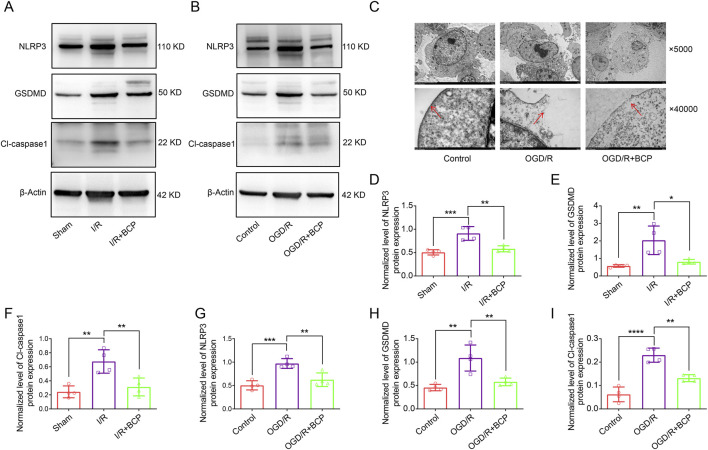
Treatment with BCP mitigated pyroptosis in mice with ischemic stroke and OGD/R-treated BV2 cells. **(A)** Representative Western blotting for the expression of NLRP3, GSDMD and Cl-caspase1 in mice; **(B)** Representative Western blotting for the expression of NLRP3, GSDMD and Cl-caspase1 in BV2 cells; **(C)** Representative morphological changes of BV2 cells determined by TEM; **(D–F)** Quantification of protein expression for NLRP3, GSDMD and Cl-caspase1 in mice; **(G–I)** Quantification of protein expression for NLRP3, GSDMD and Cl-caspase1 in BV2 cells. Data are expressed as mean ± SD (n = 4). ^*^P < 0.05; ^**^P < 0.01; ^***^P < 0.001; ^****^P < 0.0001.

Given that pyroptosis is a critical driver of neuroinflammation in cerebral ischemia (Geng et al., 2025), we investigated whether BCP exerts its protective effects by modulating this process. Western blotting analysis of peri-infarct cortical tissues revealed that the protein levels of key pyroptotic markers—including NLRP3, gasdermin D (GSDMD), and cleaved caspase-1, which are well-established canonical markers of NLRP3-mediated pyroptosis, were significantly elevated following I/R injury. Treatment with BCP significantly reversed the upregulation of these proteins (Figures 2A,D–F). To validate these findings *in vitro*, we utilized an oxygen-glucose deprivation/reoxygenation (OGD/R) model in BV2 microglial cells. Similarly, BCP pretreatment significantly decreased the OGD/R-induced upregulation of NLRP3, GSDMD, and cleaved caspase-1 (Figures 2B,G–I). Transmission electron microscopy (TEM) further corroborated these results, showing that the characteristic morphological features of pyroptosis, such as cell membrane rupture and cytoplasmic content release (indicated by red arrowheads), were evident in OGD/R-treated cells but were markedly alleviated by BCP treatment (Figure 2C). Collectively, these findings indicate that BCP mitigates cerebral I/R-induced cellular pyroptosis in both mice and microglial cells.

### BCP inhibits glycolysis in mice with ischemic stroke and OGD/R-treated BV2 cells

**FIGURE 3:**
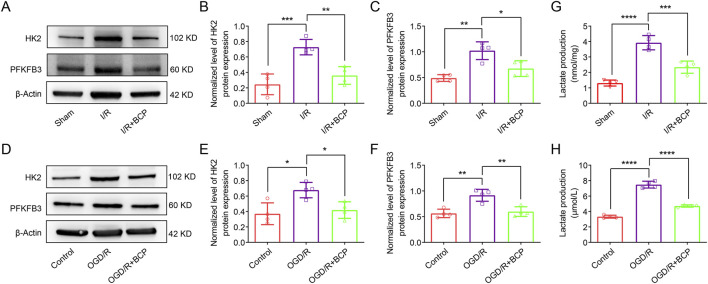
Treatment with BCP inhibited glycolysis in mice with ischemic stroke and OGD/R-treated BV2 cells. **(A)** Representative Western blotting for the expression of HK2 and PFKFB3 in mice; **(B,C)** Quantification of protein expression for HK2 and PFKFB3 in mice; **(D)** Representative Western blotting for the expression of HK2 and PFKFB3 in BV2 cells; **(E,F)** Quantification of protein expression for HK2 and PFKFB3 in BV2 cells; **(G)** Lactate levels in brain tissues of mice; **(H)** Lactate levels in BV2 cells. Data are expressed as mean ± SD (n = 4). ^*^P < 0.05; ^**^P < 0.01; ^***^P < 0.001; ^****^P < 0.0001.

Ischemic stroke is characterized by a metabolic shift towards glycolysis. We therefore assessed the impact of BCP on glycolysis. Western blotting revealed that the protein expression of key glycolytic enzymes, hexokinase 2 (HK2) and 6-phosphofructo-2-kinase/fructose-2,6-bisphosphatase 3 (PFKFB3), was significantly elevated in the peri-infarct region following I/R. This upregulation was effectively suppressed by BCP treatment (Figures 3A–C). A similar trend was observed *in vitro*, where OGD/R stimulation significantly increased HK2 and PFKFB3 levels in BV2 cells, an effect that was abrogated by BCP pretreatment (Figures 3D–F). Furthermore, measurement of lactate, a primary glycolytic byproduct, showed significant accumulation in both ischemic brain tissues and OGD/R-stimulated BV2 cells. This lactate surge was markedly attenuated by BCP administration (Figures 3G,H). Overall, these data suggest that BCP could inhibit cerebral ischemia-induced glycolysis *in vivo* and *in vitro*.

### BCP Reduces H3K9 and H3K18 lactylation in mice with ischemic stroke and OGD/R-treated BV2 cells

**FIGURE 4:**
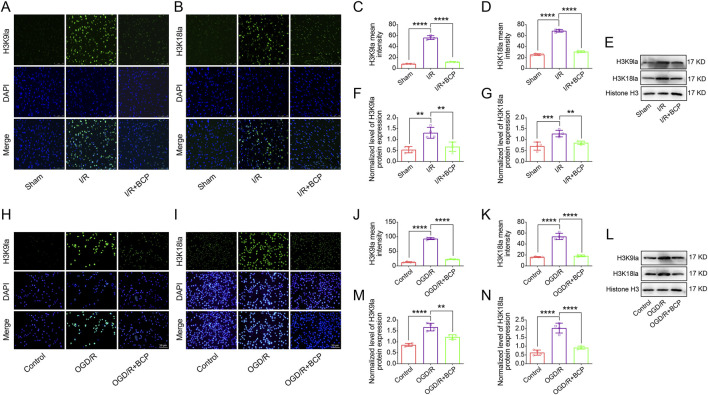
Treatment with BCP inhibited elevation of H3K9la and H3K18la in mice with ischemic stroke and OGD/R-treated BV2 cells. **(A,B)** Immunofluorescence staining of H3K9la and H3K18la in mice; **(C,D)** Mean fluorescence intensity of H3K9la and H3K18la in mice; **(E)** Representative Western blotting for the expression of H3K9la and H3K18la in mice; **(F,G)** Quantification of protein expression for H3K9la and H3K18la in mice; **(H,I)** Immunofluorescence staining of H3K9la and H3K18la in BV2 cells; **(J,K)** Mean fluorescence intensity of H3K9la and H3K18la in BV2 cells; **(L)** Representative Western blotting for the expression of H3K9la and H3K18la in BV2 cells; **(M,N)** Quantification of protein expression for H3K9la and H3K18la in BV2 cells. Data are expressed as mean ± SD (n = 4). ^**^P < 0.01; ^***^P < 0.001; ^****^P < 0.0001.

Since the accumulation of lactate could induce histone lactylation, we next investigated whether BCP influences this epigenetic modification. Immunofluorescence staining of brain sections demonstrated that the fluorescence intensity of both H3K9 lactylation (H3K9la) and H3K18 lactylation (H3K18la) was significantly increased in the I/R group compared to sham controls. Notably, BCP treatment significantly diminished these levels (Figures 4A–D). Western blotting assay confirmed that the protein levels of H3K9la and H3K18la in the peri-infarct area were upregulated after I/R but downregulated by BCP (Figures 4E–G). To extend these findings to microglia, we analyzed BV2 cells subjected to OGD/R. Both immunofluorescence and Western blotting revealed that OGD/R induced a significant increase in H3K9la and H3K18la levels, an effect that was reversed by BCP pretreatment (Figures 4H–N). Altogether, these data indicate that BCP suppresses cerebral ischemia-induced elevation of H3K9la and H3K18la.

### Lactate supplementation abolishes the neuroprotective effects of BCP

**FIGURE 5:**
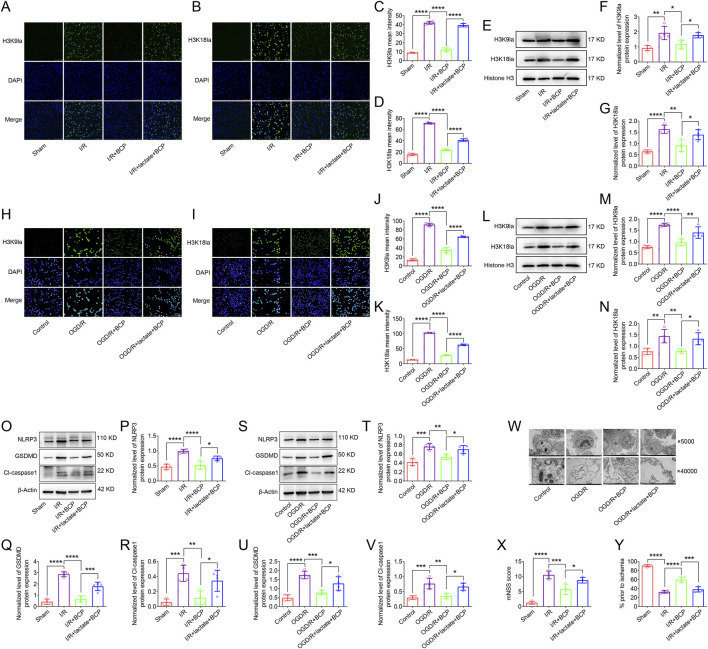
Lactate supplementation reversed the inhibitory effects of BCP on cellular pyroptosis in mice with ischemic stroke and OGD/R-treated BV2 cells. **(A,B)** Immunofluorescence staining of H3K9la and H3K18la in mice; **(C,D)** Mean fluorescence intensity of H3K9la and H3K18la in mice; **(E)** Representative Western blotting for the expression of H3K9la and H3K18la in mice; **(F,G)** Quantification of protein expression for H3K9la and H3K18la in mice; **(H,I)** Immunofluorescence staining of H3K9la and H3K18la in BV2 cells; **(J,K)** Mean fluorescence intensity of H3K9la and H3K18la in BV2 cells; **(L)** Representative Western blotting for the expression of H3K9la and H3K18la in BV2 cells; **(M,N)** Quantification of protein expression for H3K9la and H3K18la in BV2 cells; **(O)** Representative Western blotting for the expression of NLRP3, GSDMD and Cl-caspase1 in mice; **(P–R)** Quantification of protein expression for NLRP3, GSDMD and Cl-caspase1 in mice; **(S)** Representative Western blotting for the expression of NLRP3, GSDMD and Cl-caspase1 in BV2 cells; **(T–V)** Quantification of protein expression for NLRP3, GSDMD and Cl-caspase1 in BV2 cells; **(W)** Representative morphological changes of BV2 cells determined by TEM; **(X)** Analysis of mNSS scores in each group; **(Y)** Analysis of rotarod performance in each group. Data are expressed as mean ± SD (n = 4). ^*^P < 0.05; ^**^P < 0.01; ^***^P < 0.001; ^****^P < 0.0001.

As the predominant end product of glycolysis, lactic acid acts as a substrate for histone lactylation, which in turn regulates the expression of downstream inflammation-related genes. To establish a causal link between histone lactylation and BCP’s mechanism of action, we co-administered sodium lactate with BCP. Immunofluorescence and Western blotting demonstrated that while BCP treatment significantly decreased H3K9la and H3K18la levels post-stroke, concurrent lactate supplementation effectively reversed these inhibitory effects in both mice and BV2 cells (Figures 5A–N). Functionally, the suppressive effects of BCP on pyroptosis-related proteins (NLRP3, GSDMD, cleaved caspase-1) in the ischemic penumbra was abolished by lactate co-treatment (Figures 5O–R). Similarly, *in vitro* assays showed that lactate supplementation negated the ability of BCP to inhibit OGD/R-induced pyroptosis in BV2 cells (Figures 5S–V). TEM analysis further confirmed that the protective morphological changes induced by BCP were compromised in the presence of exogenous lactate (Figure 5W). Finally, behavioral assessments revealed that while BCP significantly alleviated neurological deficits and improved rotarod performance, co-administration of lactate significantly weakened this neuroprotection (Figures 5X,Y). Together, lactate supplementation effectively reversed the inhibitory effects of BCP on H3K9la and H3K18la levels and abolished its suppression of pyroptosis-related proteins, demonstrating that the neuroprotective effects of BCP are dependent on the inhibition of histone lactylation.

### BCP suppresses histone lactylation and pyroptosis in OGD/R-treated primary microglia

**FIGURE 6:**
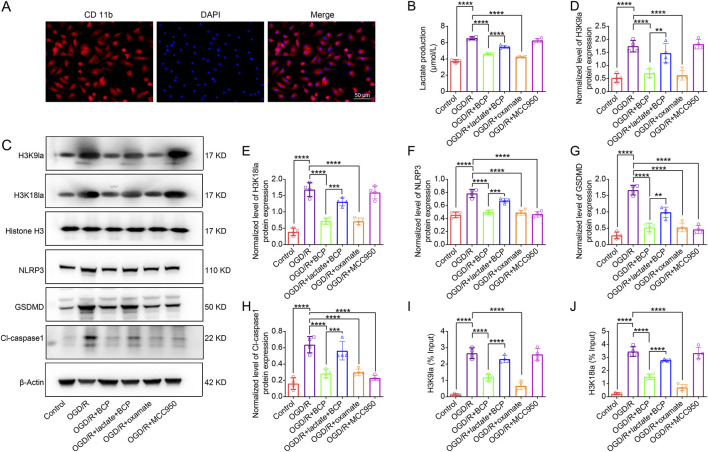
Validation of BCP-mediated inhibition of histone lactylation and pyroptosis in primary microglia. **(A)** Representative immunofluorescence staining of primary microglia using CD11 b antibody, scale bar = 50 μm; **(B)** Lactate levels in primary microglia; **(C)** Representative Western blotting for the expression of H3K9la, H3K18la, NLRP3, GSDMD and Cl-caspase1 in primary microglia; **(D–H)** Quantification of protein expression for H3K9la, H3K18la, NLRP3, GSDMD and Cl-caspase1 in primary microglia; **(I)** Enrichment of H3K9la at NLRP3 promoter in primary microglia, analyzed using Chip-PCR; **(J)** Enrichment of H3K18La at NLRP3 promoter in primary microglia, analyzed using Chip-PCR. Data are expressed as mean ± SD (n = 4). ^**^P < 0.01; ^***^P < 0.001; ^****^P < 0.0001.

Next, we validated these findings in primary microglia. The purity of primary microglia was confirmed by CD11 b immunofluorescence staining (over 90%), sufficient for subsequent experiments (Figure 6A). Consistent with the results obtained from BV2 cells, OGD/R injury significantly increased lactate accumulation and induced the expression of H3K9la, H3K18la, and pyroptosis-related proteins (NLRP3, GSDMD, and cleaved caspase-1) in primary microglia. BCP treatment markedly reversed these abnormal changes; whereas exogenous lactate supplementation partially attenuated the regulatory effects of BCP. In addition, the LDHA inhibitor oxamate simultaneously decreased lactate levels and downregulated H3K9la, H3K18la, and pyroptosis-related protein levels; in contrast, the NLRP3 inflammasome inhibitor MCC950 reduced pyroptosis-related protein levels without significantly affecting lactate levels or H3K9la and H3K18la protein expression (Figures 6B–H). To further clarify the role of histone lactylation modification in NLRP3 gene regulation, we employed Chip-PCR to detect the enrichment levels of H3K9la and H3K18la at the NLRP3 promoter region. As shown in Figures 6I,J, OGD/R injury significantly increased the enrichment of H3K9la and H3K18la at the NLRP3 promoter; BCP treatment significantly reduced this enrichment, and lactate supplementation partially inhibited the regulatory action of BCP. Additionally, oxamate markedly suppressed the enrichment, whereas MCC950 had no significant effect. In summary, these studies provide more physiologically relevant experimental evidence for the involvement of lactate-mediated histone lactylation in the regulation of microglial pyroptosis.

## Discussion

In the present study, we established a mouse model of focal cerebral ischemia/reperfusion (I/R) and an *in vitro* oxygen-glucose deprivation/reoxygenation (OGD/R) model in BV2 microglial cells and primary microglia to elucidate the neuroprotective mechanisms of β-caryophyllene (BCP). Our *in vivo* data demonstrate that BCP administration significantly reduced cerebral infarct volume, ameliorated neurological deficits, and improved motor coordination. Mechanistically, we identified that BCP exerts these effects by suppressing cellular pyroptosis, a process driven by the inhibition of H3K9 and H3K18 histone lactylation.

As a naturally occurring bicyclic sesquiterpene, BCP has garnered significant attention for its therapeutic potential in neurological disorders. Accumulating evidence supports its regulatory effects on ferroptosis, apoptosis, and inflammation, thereby conferring protection against cerebral I/R injury (Yokubaitis et al., 2021; Hu et al., 2022). Our previous work further demonstrated that BCP mitigates ischemic stroke-induced white matter damage (Xin et al., 2024). Building on these foundations, the current study utilized complementary *in vivo* and *in vitro* models to validate the beneficial effects of BCP. Consistent with prior literature, we observed that ischemic insult led to significant tissue damage and functional impairment, both of which were effectively attenuated by BCP treatment.

Cerebral I/R injury is intrinsically linked to neuroinflammation and microglial activation. Central to this pathology is pyroptosis, a pro-inflammatory form of programmed cell death primarily orchestrated by the NLRP3 inflammasome (Li X. et al., 2024). Activation of this multiprotein complex facilitates the autoactivation of caspase-1, which subsequently cleaves gasdermin D (GSDMD) to execute pyroptosis and promotes the maturation of pro-inflammatory cytokines, including IL-1β and IL-18 (Du et al., 2025). In this study, we observed a substantial upregulation of NLRP3, GSDMD, and cleaved caspase-1 in both ischemic mouse brains and OGD/R-stimulated BV2 cells. Furthermore, transmission electron microscopy revealed characteristic pyroptotic morphology—cellular swelling and membrane rupture—in OGD/R-treated cells, which was mitigated by BCP pretreatment. These findings robustly indicate that BCP suppresses NLRP3-mediated pyroptosis following cerebral ischemic injury.

Ischemic stroke precipitates a metabolic crisis, forcing a shift from oxidative phosphorylation to anaerobic glycolysis. This metabolic reprogramming results in insufficient ATP production and the aberrant accumulation of lactate within the brain parenchyma, a phenomenon well-documented in both clinical (Zhou et al., 2024) and preclinical settings (Pan et al., 2025). In the present study, we found that BCP significantly reduced the protein levels of key glycolytic enzymes (HK2, PFKFB3) and lactate production in ischemic brain tissues and BV2 cells after OGD/R treatment.

As the major metabolic product of glycolysis, lactate can directly amplify the inflammatory process and further aggravate tissue damage when abnormally accumulated (Fang et al., 2024). In 2019, Zhang et al. (Zhang et al., 2019) first described histone lactylation, a novel post-translational modification where lactate serves as a substrate to modify histone lysine residues, thereby directly regulating gene transcription. Emerging evidence has linked this epigenetic modification to the transcriptional activation of inflammatory genes. For instance, glycolytic reprogramming has been shown to exacerbate inflammatory responses in the kidney and lung by enhancing histone lactylation (Li J. et al., 2024; Wang et al., 2024c). Specifically regarding pyroptosis, several studies have established a critical role for histone lactylation. You et al. reported that silica-induced lactate accumulation drives NLRP3-dependent pyroptosis via histone lactylation in macrophages (You et al., 2024), a finding mirrored in astrocytic models of bilirubin encephalopathy (Li et al., 2025) and selenium deficiency-induced gallbladder inflammation (An et al., 2026). Similarly, dexamethasone has been shown to suppress NLRP3-mediated pyroptosis by inhibiting glycolysis and lactylation in asthmatic models (Chen et al., 2024). In alignment with these reports, our present data demonstrated that BCP treatment led to a marked decrease in the ischemic penumbra of mice and OGD/R-treated BV2 cells. Crucially, the neuroprotective effects of BCP—including the suppression of pyroptosis and improvement of neurological function—were effectively reversed by exogenous lactate supplementation. These findings confirm that BCP exerts its protective effects by targeting the “lactate-histone lactylation-pyroptosis” axis, highlighting the critical role of this pathway in the pathophysiology of ischemic brain injury.

While BV2 cells serve as a widely used immortalized microglial cell line with high experimental controllability, they do not fully recapitulate the phenotypic characteristics of primary microglia. To compensate for the limitations and enhance the physiological relevance of our findings, we performed further validation experiments in primary microglia. Similar to the results from BV2 cells, OGD/R also significantly increased lactate accumulation and upregulated the expression of H3K9la, H3K18la, and pyroptosis-related proteins in primary microglia, whereas BCP intervention markedly reversed these abnormal alterations. It should be noted that exogenous lactate supplementation partially attenuated the inhibitory effects of BCP on lactate, histone lactylation, and pyroptosis-related proteins. This rescue experiment corroborated those observations in BV2 cells, highlighting the pivotal role of lactate as an intermediary molecule connecting metabolism with histone lactylation in BCP-mediated neuroprotection. More importantly, the LDHA inhibitor oxamate simultaneously decreased lactate levels and downregulated H3K9la, H3K18la, and pyroptosis-related protein levels; in contrast, the NLRP3 inflammasome inhibitor MCC950 only reduced pyroptosis-related protein levels without affecting lactate levels or H3K9la and H3K18la protein expression. Chip-PCR results further demonstrated that OGD/R injury significantly increased the enrichment of H3K9la and H3K18la at the NLRP3 promoter region, and BCP intervention reduced this enrichment; lactate supplementation partially attenuated the transcriptional regulatory role of BCP. Oxamate treatment significantly decreased histone lactylation enrichment at the NLRP3 promoter region, whereas MCC950 had no significant regulatory effect on this epigenetic modification, providing a more physiologically relevant experimental basis for the involvement of the lactate-histone lactylation-pyroptosis axis in microglial pathophysiology.

Although the above pharmacological evidence has established a relatively complete mechanism from multiple perspectives, this study primarily relies on pharmacological interventions, which makes it difficult to establish causal relationships regarding the lactate-lactylation-pyroptosis axis. Pharmacological agents inherently possess off-target effects; for instance, oxamate may exert non-specific inhibitory effects on other LDH isoenzymes. Furthermore, *in vitro* culture systems cannot fully recapitulate the complex *in vivo* neuroinflammatory microenvironment. This study mainly explores the histone lactylation-pyroptosis regulatory axis based on *in vitro* experimental evidence from microglia. Nevertheless, the regulatory effects of BCP on histone lactylation may involve synergistic actions of multiple cell types such as neurons and astrocytes. Although microglia serve as one of the key cellular targets mediating the neuroprotective effects of BCP, their relative contribution and crosstalk with other cell types remain to be further elucidated. Alternatively, this study only focused on H3K9la and H3K18la, and the regulatory effects of BCP on other histone lactylation sites (such as H4K12la) remain to be further explored. Follow-up studies will adopt genetic approaches including siRNA targeting histone lactylation writers and erasers, CRISPR-based gene editing, as well as microglia-specific conditional knockout models, to clarify the causal regulatory relationship between histone lactylation and NLRP3 transcriptional activation. In the meantime, we will systematically clarify the specific contributions of various cell types to the effects of BCP by optimizing antibody combinations and applying multicolor immunofluorescence.

## Conclusion

**FIGURE 7:**
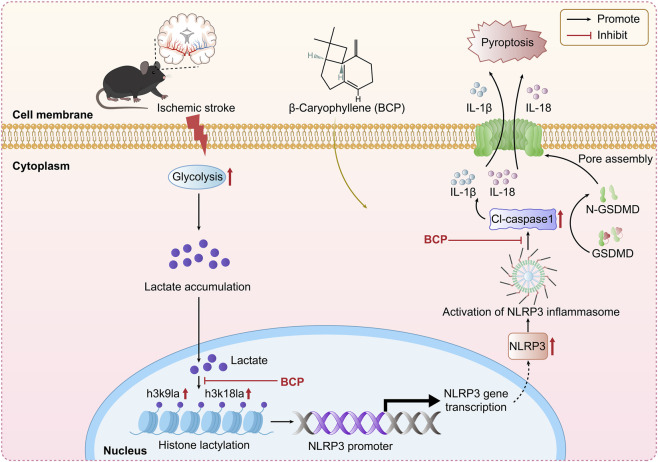
Schematic diagram of the molecular mechanism underlying the neuroprotective effects of BCP.

In conclusion, this study demonstrates that BCP exerts neuroprotective effects against ischemic stroke by inhibiting H3K9 and H3K18 lactylation, thereby suppressing NLRP3-mediated cellular pyroptosis (Figure 7). These data suggest that BCP is a promising therapeutic candidate for the prevention and treatment of cerebral ischemic injury, providing a solid theoretical basis for future clinical translation.

## Data Availability

The original contributions presented in the study are included in the article/Supplementary Material, further inquiries can be directed to the corresponding author.
