# Supplementary figures and images for: β-Caryophyllene protects against ischemic stroke by inhibiting H3K9 and H3K18 lactylation-mediated cellular pyroptosis
Source: Front Pharmacol. 2026 Jun 17;17:1840441. doi: 10.3389/fphar.2026.1840441 (PMC13318654; doi:10.3389/fphar.2026.1840441)

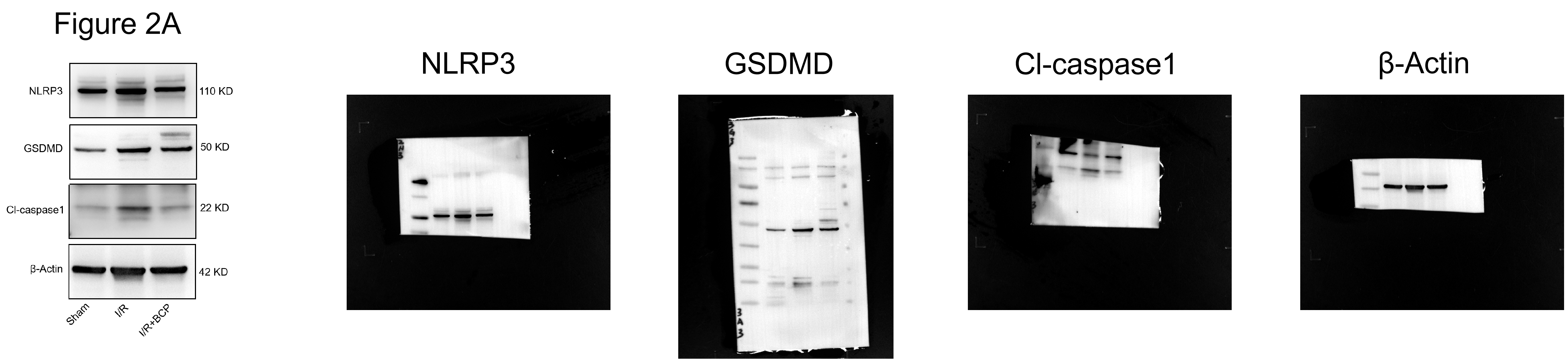

Supplement: Supplementary file 1 [file DataSheet1.zip › Original western blotting images/Figure 2A.tif]

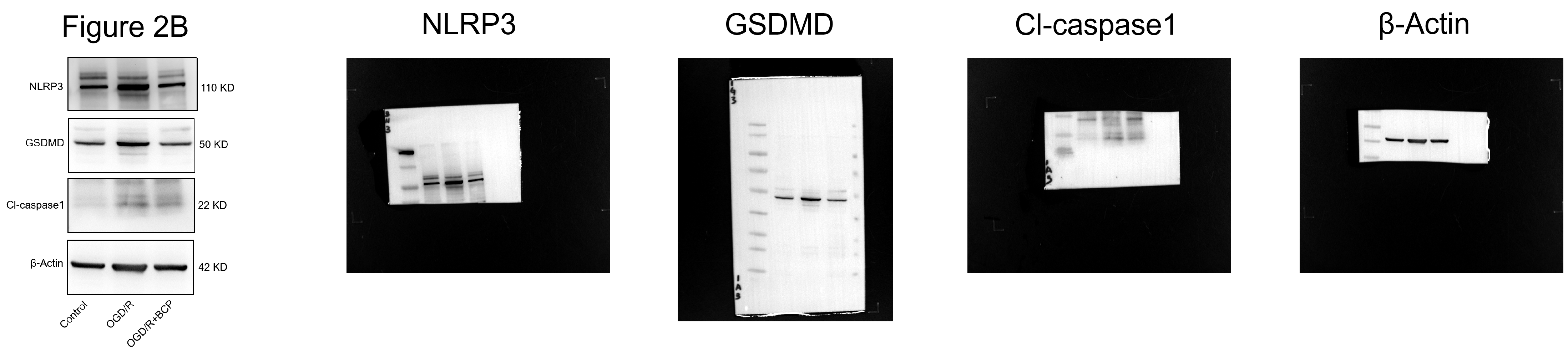

Supplement: Supplementary file 1 [file DataSheet1.zip › Original western blotting images/Figure 2B.tif]

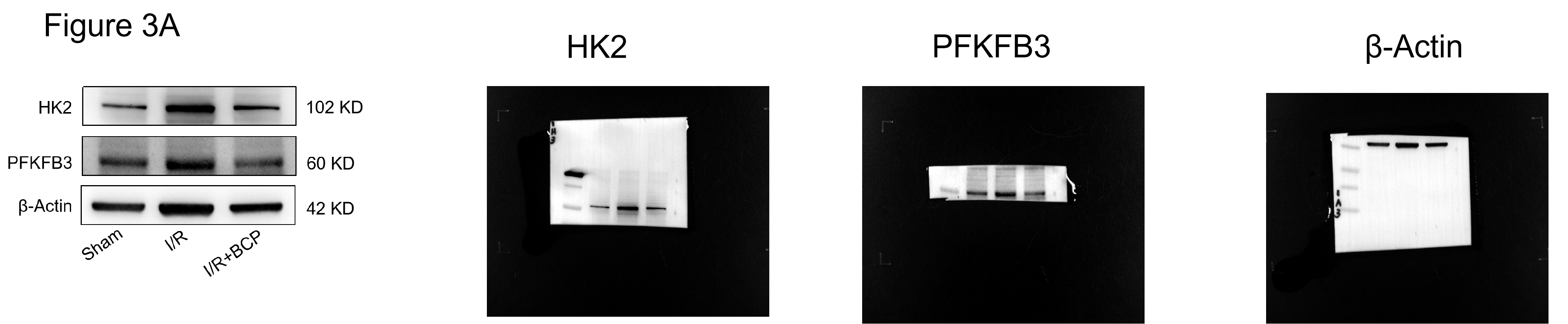

Supplement: Supplementary file 1 [file DataSheet1.zip › Original western blotting images/Figure 3A.tif]

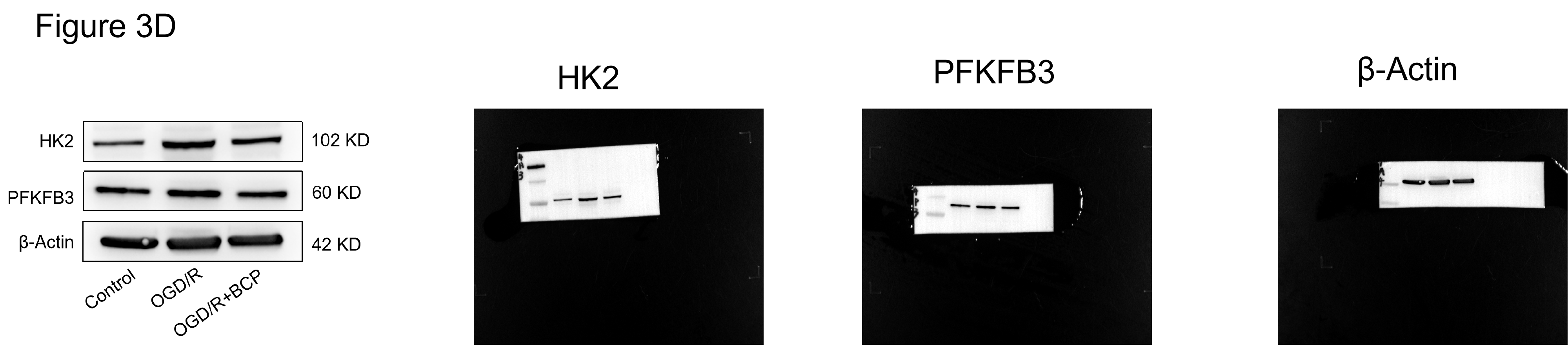

Supplement: Supplementary file 1 [file DataSheet1.zip › Original western blotting images/Figure 3D.tif]

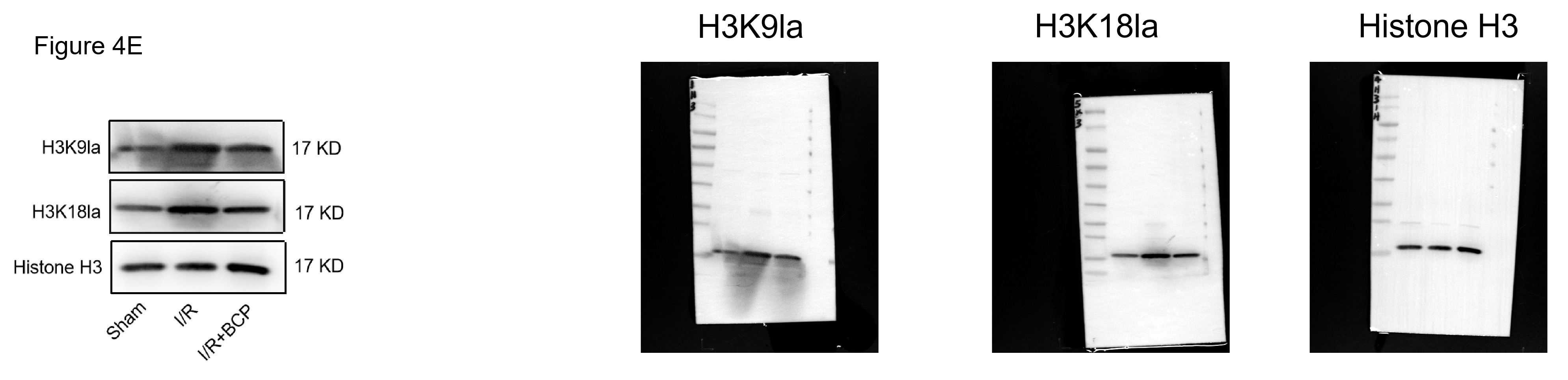

Supplement: Supplementary file 1 [file DataSheet1.zip › Original western blotting images/Figure 4E.tif]

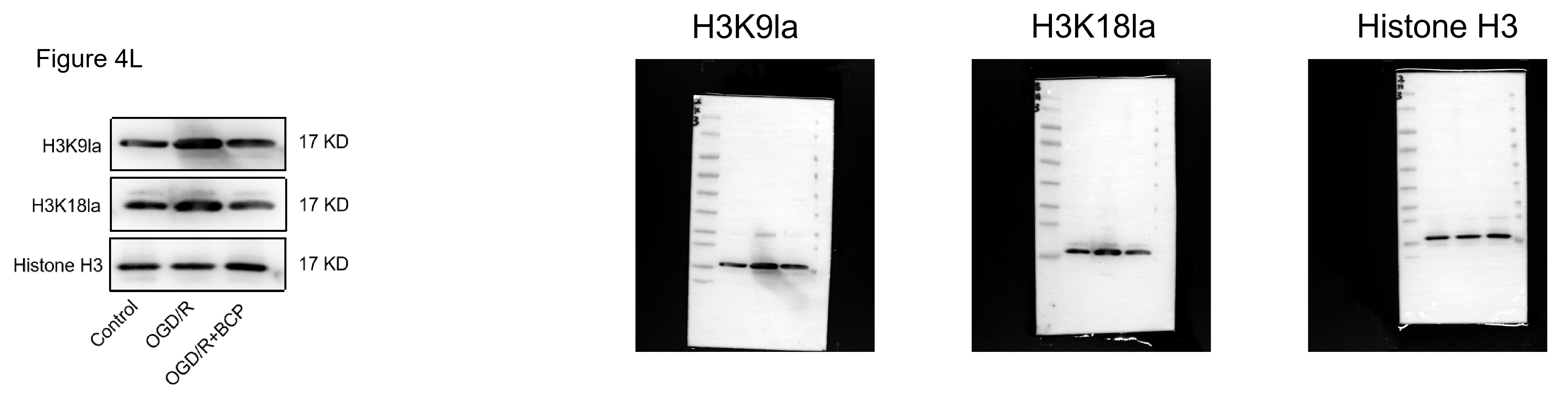

Supplement: Supplementary file 1 [file DataSheet1.zip › Original western blotting images/Figure 4L.tif]

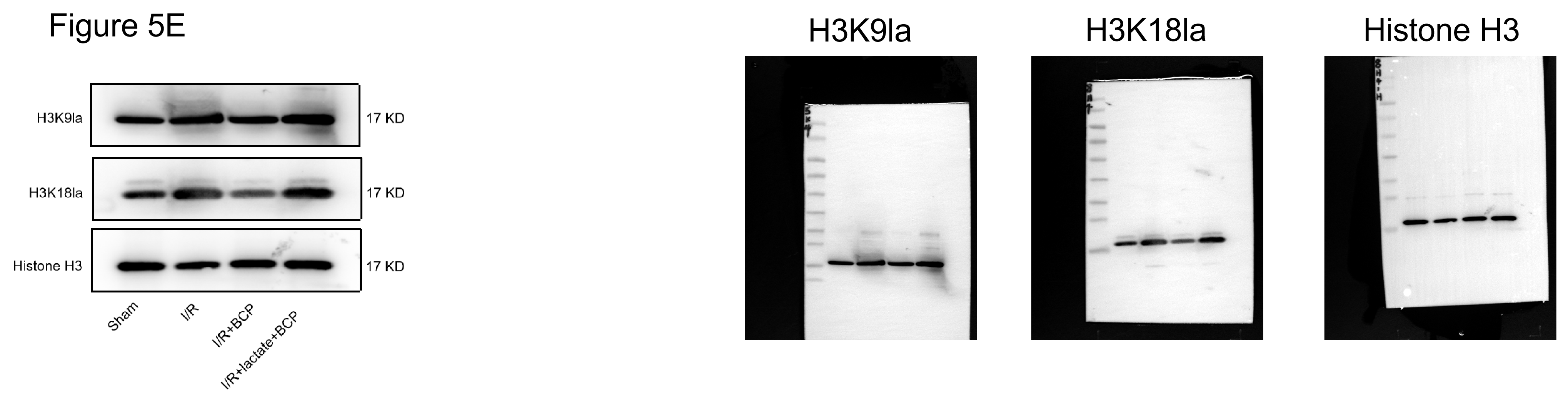

Supplement: Supplementary file 1 [file DataSheet1.zip › Original western blotting images/Figure 5E.tif]

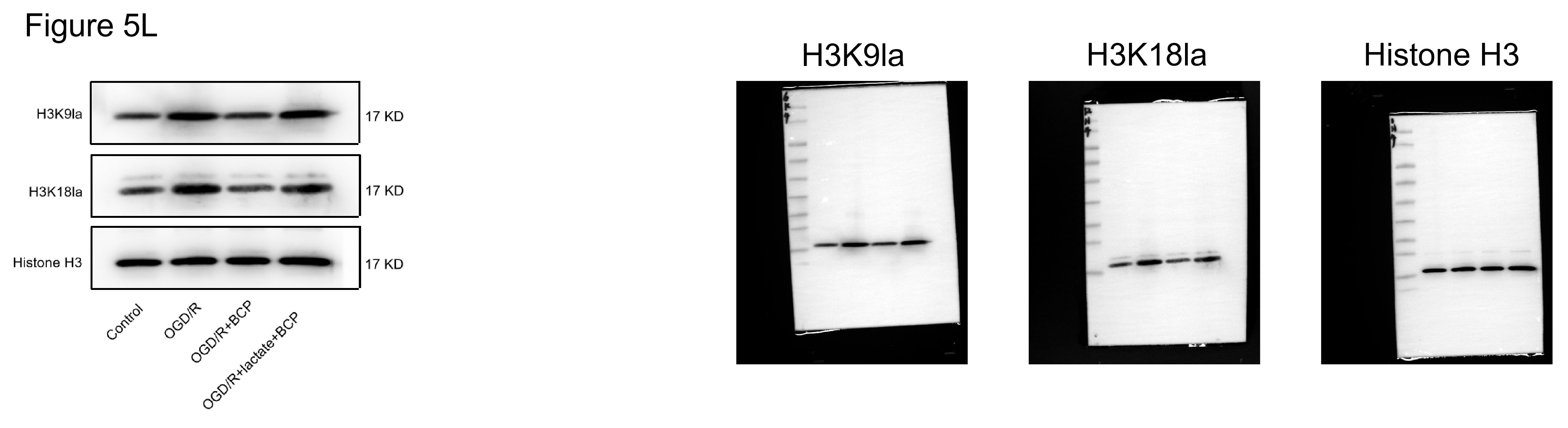

Supplement: Supplementary file 1 [file DataSheet1.zip › Original western blotting images/Figure 5L.tif]

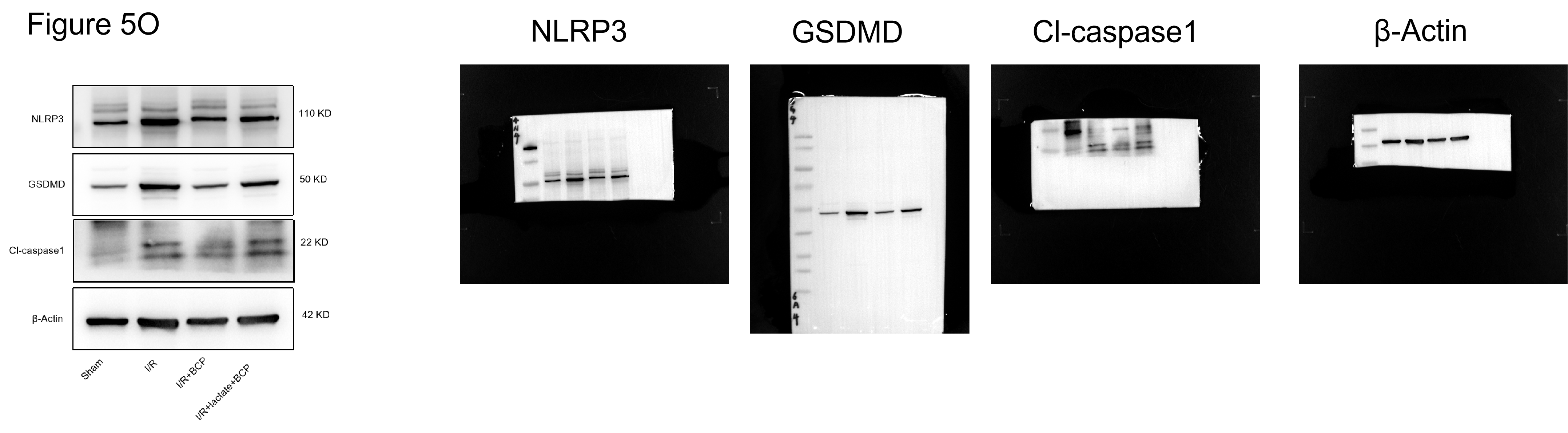

Supplement: Supplementary file 1 [file DataSheet1.zip › Original western blotting images/Figure 5O.tif]

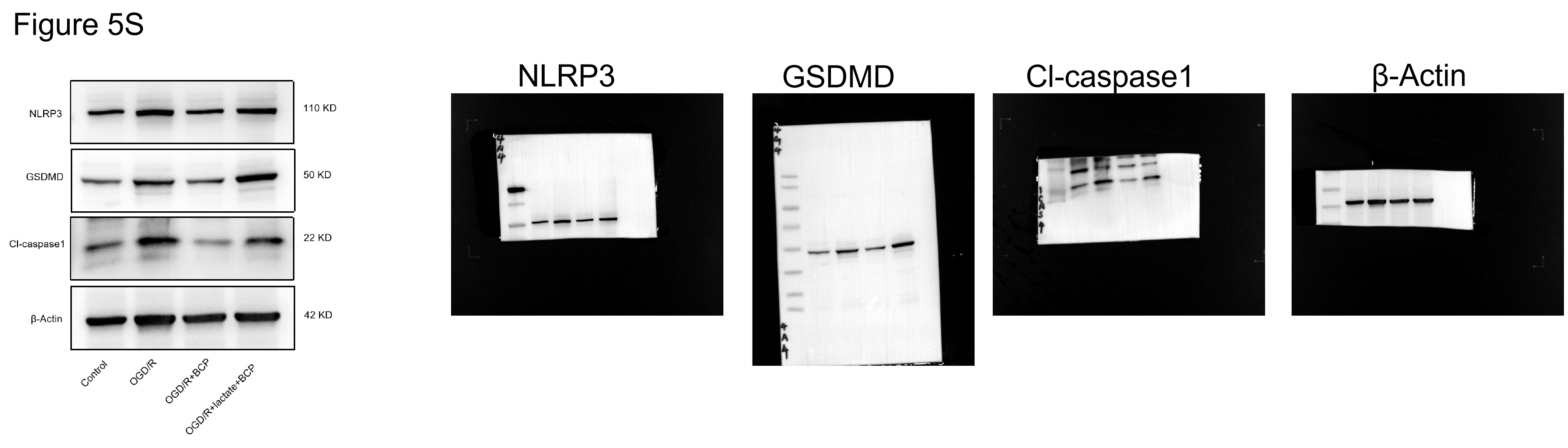

Supplement: Supplementary file 1 [file DataSheet1.zip › Original western blotting images/Figure 5S.tif]
